# Sphingosine-1-phosphate promotes liver fibrosis in metabolic dysfunction-associated steatohepatitis

**DOI:** 10.1371/journal.pone.0303296

**Published:** 2024-05-16

**Authors:** Yosuke Osawa, Hironari Kawai, Keigo Nakashima, Yuichi Nakaseko, Daisuke Suto, Keisuke Yanagida, Tomomi Hashidate-Yoshida, Taizo Mori, Sachiyo Yoshio, Takaaki Ohtake, Hideo Shindou, Tatsuya Kanto

**Affiliations:** 1 Departments of Gastroenterology, International University of Health and Welfare Hospital, Tochigi, Japan; 2 Department of Liver Diseases, The Research Center for Hepatitis and Immunology, National Center for Global Health and Medicine, Chiba, Japan; 3 Department of Surgery, The Jikei University School of Medicine, Tokyo, Japan; 4 Departments of Surgery, International University of Health and Welfare Hospital, Tochigi, Japan; 5 Departments of Lipid Life Science, National Center for Global Health and Medicine, Tokyo, Japan; 6 Departments of Medical Lipid Science, Graduated Scholl of Medicine, The University of Tokyo, Tokyo, Japan; Universite Paris Diderot-Paris7 - Batiment des Grands Moulins, FRANCE

## Abstract

**Aim:**

Metabolic dysfunction-associated steatohepatitis (MASH) is one of the most prevalent liver diseases and is characterized by steatosis and the accumulation of bioactive lipids. This study aims to understand the specific lipid species responsible for the progression of liver fibrosis in MASH.

**Methods:**

Changes in bioactive lipid levels were examined in the livers of MASH mice fed a choline-deficient diet (CDD). Additionally, sphingosine kinase (SphK)1 mRNA, which generates sphingosine 1 phosphate (S1P), was examined in the livers of patients with MASH.

**Results:**

CDD induced MASH and liver fibrosis were accompanied by elevated levels of S1P and increased expression of SphK1 in capillarized liver sinusoidal endothelial cells (LSECs) in mice. SphK1 mRNA also increased in the livers of patients with MASH. Treatment of primary cultured mouse hepatic stellate cells (HSCs) with S1P stimulated their activation, which was mitigated by the S1P receptor (S1PR)2 inhibitor, JTE013. The inhibition of S1PR2 or its knockout in mice suppressed liver fibrosis without reducing steatosis or hepatocellular damage.

**Conclusion:**

S1P level is increased in MASH livers and contributes to liver fibrosis via S1PR2.

## Introduction

Metabolic dysfunction-associated steatotic liver disease (MASLD) is a component of the metabolic syndrome and encompasses a range of liver disorders, including simple steatosis and Metabolic dysfunction-associated steatohepatitis (MASH), which consist of lobular inflammation, hepatocellular ballooning, and apoptosis, leading to liver fibrosis [1–3]. Although simple steatosis is generally benign, patients with liver fibrosis have shorter survival than those without fibrosis [4]. Hepatic steatosis occurs when the import and synthesis of lipids exceed their export or catabolism in the hepatocytes. Excessive exposure of hepatocytes to free fatty acids (FFAs) or carbohydrates from the gut is the primary cause of hepatic steatosis. In addition to steatosis, several other factors contribute to the progression of steatosis to MASH and fibrosis. Previous studies employing lipidomics analysis of MASLD livers have demonstrated abnormal lipid composition and accumulation of bioactive lipids, such as ceramides, FFAs, cholesterol, platelet-activating factor (PAF), and eicosanoids, leading to lipotoxicity, hepatocyte injury, and liver inflammation [5,6]. However, the roles of bioactive lipids in liver fibrosis, particularly their impact on hepatic stellate cells (HSCs) activation, remains unclear.

Ceramides are deacylated by ceramidases and converted to sphingosine, which is further phosphorylated by sphingosine kinase (SphK), leading to the generation of sphingosine-1-phosphate (S1P). S1P functions as a bioactive lipid and protects hepatocytes from the cytotoxic effects of tumor necrosis factor (TNF)-α [7]. Additionally, S1P promotes macrophage migration and regulates Timp-1 expression in hepatic stellate cells [8]. S1P is involved in regulating glucose and lipid metabolism in hepatocytes via AKT-mediated signaling [9]. Moreover, S1P has been shown to contributes to liver fibrosis through the S1P receptor (S1PR)2 in various animal models, including bile duct ligation [10,11], carbon tetrachloride-induced liver injury [12,13], and inferior vena cava ligation [14]. In a high-fructose, saturated fat, and cholesterol-induced MASH model, treatment with the S1PR1 antagonist, FTY720, ameliorated liver injury, inflammation, and fibrosis by reducing hepatic macrophage accumulation [15].

Under pathological conditions, liver sinusoidal endothelial cells (LSECs) undergo capillarization, characterized by the loss of fenestrae and the development of a basement membrane, which promotes angiogenesis and is associated with the progression of liver fibrosis [14,16,17]. Angiopoietins (ANGP) play a crucial role in regulating angiogenesis. Elevated levels of serum ANGP-2 have been observed in patients with MASH, and ANGP-2 inhibition has been shown to improve fibrosis in a mouse model of MASH [18]. Endothelial cells are a source of S1P [14,19].

In this study, we aimed to elucidate the changes in bioactive lipids and their roles in MASH-induced liver fibrosis using an animal model. We used a well-established MASH mouse model by feeding mice a choline-deficient diet (CDD). These data suggest that S1P levels are increased in the MASH liver and that S1P contributes to liver fibrosis through the activation of S1PR2.

## Materials and methods

### Study approval

This study was conducted in accordance with the Helsinki Declaration. This study was approval by the institutional research ethics committees at NCGM Kohnodai hospital (NCGM-G-003232-00) and IUHW hospital (20-B-447). Written informed consent or informed assent was obtained from patients or legal guardians. All experiments for animals were conducted in accordance with institutional guidelines (Guide for the Care and Use of Laboratory Animals prepared by the National Academy of Sciences) and the protocol was approved by the Animal Research Committee of National Center for Global Health and Medicine (NO. 20555 and 20056). In accordance with the provisions of Article 8 of the Regulations for Gene Recombination Experiments at the National Center for Global Health and Medicine, the necessary application was submitted and approved prior to conducting the genetic engineering experiments (2-D-074, 2-D-075, and 2-D-076).

### Patients

Liver samples from patients with MASLD or healthy controls were collected from individuals who had undergone liver biopsy or partial hepatectomy. The recruitment period extended from March 1, 2021 to March 24, 2023. Excess tissue not intended for diagnostic purposes was utilized. Non-tumor tissues were used for analysis. The patients were pathologically diagnosed with either fatty liver or MASH. Clinical data were obtained from their electronic clinical records. All patients tested negative for hepatitis B surface antigen (HBsAg). Among the patients, three tested positive for hepatitis C virus antibody (HCVAb), but HCV RNA was not detected. None of the patients met the criteria for autoimmune hepatitis based on the revised original score for autoimmune hepatitis. Additionally, all patients tested negative for anti-mitochondrial M2 antibody (AMAM2). Alcohol consumption in these patients was below 20 g of ethanol per day. Liver stiffness measurement was performed using shear wave elastography (LOGIQ S8, GE Healthcare). Liver fibrosis was evaluated using a previously reported classification system [20]. F0 was defined as the absence of liver fibrosis.

### Animal treatments

MASH was induced by feeding 5-week-old male animals with a CDD (Research Diets, Inc. New Brunswick, NJ, USA) for 8 weeks. This diet impairs the synthesis of phosphatidylcholine, an essential component of the outer phospholipid layer lipoproteins. As a result, there is a reduction in the secretion of liver triglyceride as very low-density lipoprotein, leading to the accumulation of triglycerides in the liver [21]. Some mice were administrated with the S1PR2 inhibitor JTE013 (0.1 mg/mouse in 5 μL dimethyl sulfoxide [DMSO] + 95 μL corn oil, intraperitoneally twice a week [CAY10009458, Cayman Chemical, Ann Arbor, MI, USA]). Control mice were fed a normal diet (ND) (12.6% of calories from fat; CE-2, CLEA Japan, Tokyo, Japan). For the wild-type mice, animals were randomized to allocate experimental units to control and treatment groups. The mice were housed under a 12/12-hour light/dark cycle, maintained in a temperature-controlled (24°C ± 2°C) setting, and provided free access to food and water. At the end of study period, the animals were deprived of food for 18 hours. After recording body weight, the mice were anesthetized via intraperitoneal administration of a mixture of medetomidine, midazolam, and butorphanol, and subsequently humanely euthanized by blood withdrawal. At least six animals were used in one group, and a total of 81 mice were used. The required number was calculated and determined based on α at 5% and β at 20%. All mice that completed the treatment were included in the analysis. The liver was immediately removed, weighted, and washed in ice-cold PBS. A part of the dissected liver tissue was frozen in liquid nitrogen, and another part of the liver was fixed with 10% formalin or embedded in optimal cutting temperature compound (Sakura Finetek Japan, Tokyo, Japan) for histological analysis. We made conscientious efforts to minimize any potential suffering experienced by the animals involved in this study. This included providing appropriate anesthesia and analgesia during procedures, ensuring comfortable housing conditions, and adhering to approved protocols for handling and care. Additionally, all procedures were conducted in accordance with institutional guidelines and ethical standards to prioritize the welfare of the animals.

### Statistical analysis

Data are presented as means ± SD of at least three independent experiments. Group comparisons were performed using the two-tailed Mann-Whitney U test or Student t-test. Correlation analysis was conducted using Spearman’s rank correlation coefficients. A p-value of less than 0.05 was considered statistically significant. The identification of outliers was performed using the ROUT method. Tests for normality and homoscedasticity were conducted, and appropriate statistical analysis methods were employed. Statistical analyses were performed using GraphPad Prism version 5 (Graph Pad Software Inc., San Diego, CA, USA).

### Other experimental procedures

Other experimental procedures are described in the Supplementary Experimental Procedures. These include animals, cell isolation, cell culture and treatment, histological analysis, measurement of bioactive lipids, ANGP2 measurement, western blotting, quantitative real-time reverse transcription (qRT)-PCR, alanine aminotransferase (ALT) and FFA measurement, measurement of triglycerides and phosphatidyl choline in the livers, hydroxyproline measurement, and data analysis.

## Results

### Changes in bioactive lipids in MASH liver

A well-established MASH mouse model using CDD feeding was utilized to investigate alterations in bioactive lipids in MASH livers. The 8-week CDD feeding resulted in the development of hepatitis (S1A Fig), increased liver weight (S1B Fig), and triglyceride accumulation in the livers, accompanied by a decrease in total phosphatidylcholine levels (S1C Fig). Additionally, MASH livers exhibited liver fibrosis and HSC activation, as evidenced by Sirius Red staining, hydroxyproline content (S1D Fig), and increased expression of the HSC activation marker α-smooth muscle actin (αSMA) (S1E Fig). Hepatocellular damage was observed in MASH mice (S1F Fig), and the mRNA expression levels of the inflammatory cytokine TNF-α (S1G Fig) and chemokines (S1 Table) were elevated in the MASH livers. Thus, CDD effectively induced MASH in mice.

**Fig 1:**
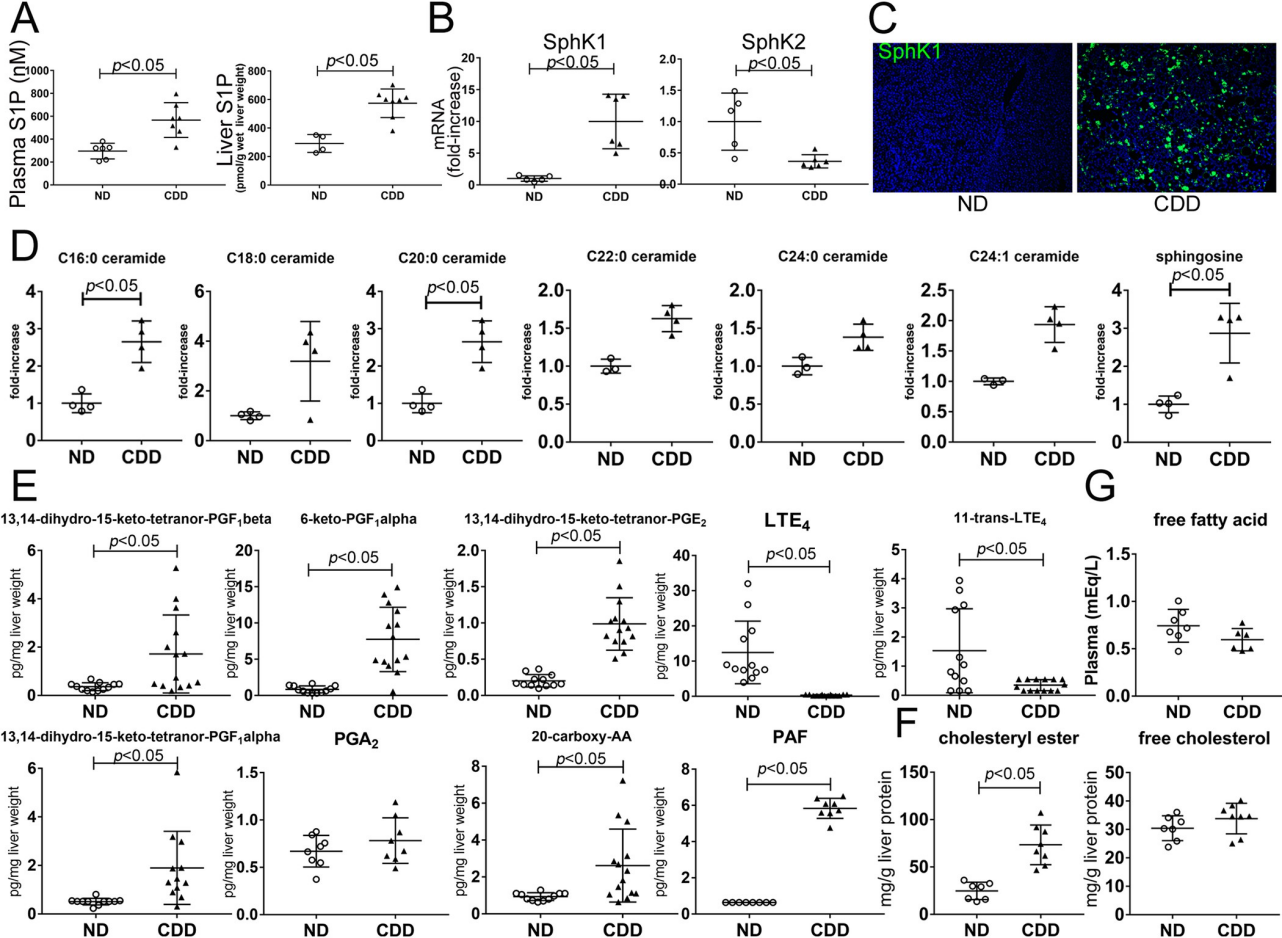
Changes in bioactive lipids in MASH liver. C57BL/6J male mice were fed a normal diet or CDD for 8 weeks and were euthanized. (A) S1P levels in the plasma or liver were measured by ELISA or LC/MS/MS. (B) mRNA expression of SphK1 and SphK2 in the livers was measured using **quantitative real-time RT-PCR.** (C) Expression of SphK1 was examined by immunohistochemistry with FITC-conjugated anti-SphK1 antibody (original magnification; ×100). The levels of ceramides and sphingosine (D), eicosanoids and PAF (E) in the livers were determined by LC/MS/MS. (F) The levels of cholesterol ester and free cholesterol in the liver were measured. (G) Plasma FFA levels were determined. Results are representative of at least 3 independent experiments. Results are shown as the means ± SD. *P < 0.05 based on a 2-tailed Student’s t-test. ND; normal diet. CDD; choline-deficient diet.

We examined the changes in bioactive lipids in MASH livers. In MASH mice, S1P levels were increased in both plasma and livers compared to those in control diet-fed mice (Fig 1A) accompanied by increased SphK1 expression but decreased SphK2 expression (Fig 1B and 1C). The levels of C16:0 and C20:0 ceramides, as well as sphingosine, were also increased in MASH livers (Fig 1D). Furthermore, the levels of 13,14-dihydro-15-keto-tetranor-prostaglandin (PG)F_1_β, 13,14-dihydro-15-keto-tetranor-PGF_1_α, 6-keto-PGF_1_α, 13,14-dihydro-15-keto-tetranor-PGE_2_, and PAF were increased in the MASH liver induced by CDD, while leukotriene (LT)E_4_ and 11-trans-LTE_4_ were decreased (Fig 1E). Free cholesterol, which is considered a critical lipotoxic molecule in MASH development [22], was not increased by CDD in our model. Conversely, the cholesterol ester levels increased (Fig 1F). Thus, cholesterol plays a minor role in the CDD-induced MASH model. Plasma FFA levels in MASH mice were comparable with those in control mice (Fig 1G).

These data suggest that changes in bioactive lipid levels may play a role in MASH development.

### S1P promotes liver fibrosis

**Fig 2:**
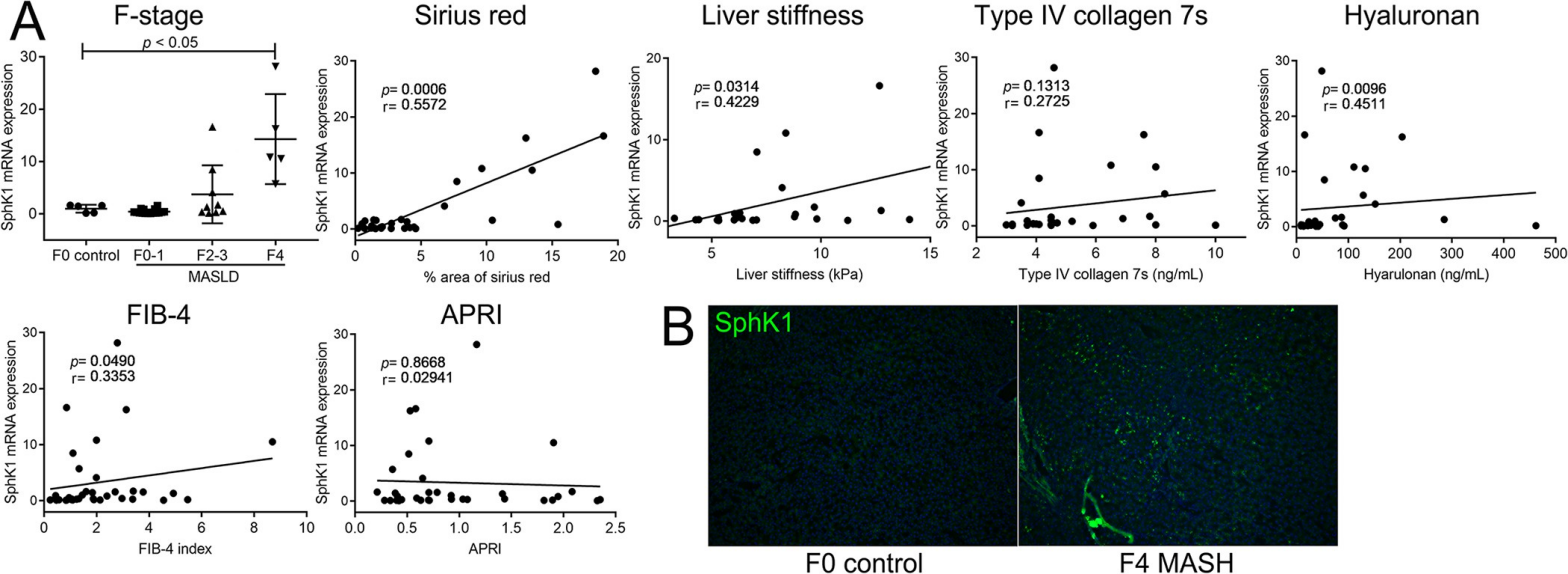
SphK1 mRNA expression in the liver of MASLD patients. (A) The levels of mRNA expression of SphK1 in the livers from MASLD patients were measured using **quantitative real-time RT-PCR and compared to those from control livers. Correlations of the SphK1 expression with the indicated values and indexes are shown. (B)** Expression of SphK1 in the cirrhotic liver from MASH patients was examined by immunohistochemistry with FITC-conjugated anti-SphK1 antibody (original magnification; ×100). Results are shown as the means ± SD. *P < 0.05 based on a 2-tailed Student’s t-test. The values for Spearman’s correlation coefficient are indicated.

To investigated whether SphK1 expression is increased in the livers of patients with MASLD, we examined the mRNA expression levels of SphK1 in MASH livers and compared them with those in normal livers obtained from liver biopsy or hepatectomy. The clinical characteristics of the patients were determined based on blood and liver samples and are summarized in S2 Table. The mRNA expression levels of SphK1 were found to be higher in the F stage 4 MASH cirrhotic livers than in the F0 control group. Furthermore, SphK1 mRNA showed a positive correlation with the percentage area of Sirius Red staining (r = 0.56) and a weak correlation with liver stiffness, hyaluronan levels, and the FIB-4 index (Fig 2A). These results indicate that SphK1 mRNA expression reflects the degree of liver fibrosis. In contrast, no correlation was observed between SphK1 mRNA expression and ALT, steatosis, HbA1C, HOMA index, and serum triglyceride levels (S2 Fig), suggesting that SphK1 mRNA expression in the liver does not reflect glucose and lipid metabolism or hepatocellular damage. Immunostaining also revealed increased protein expression of SphK1 in the cirrhotic livers of patients with MASH (Fig 2B).

**Fig 3:**
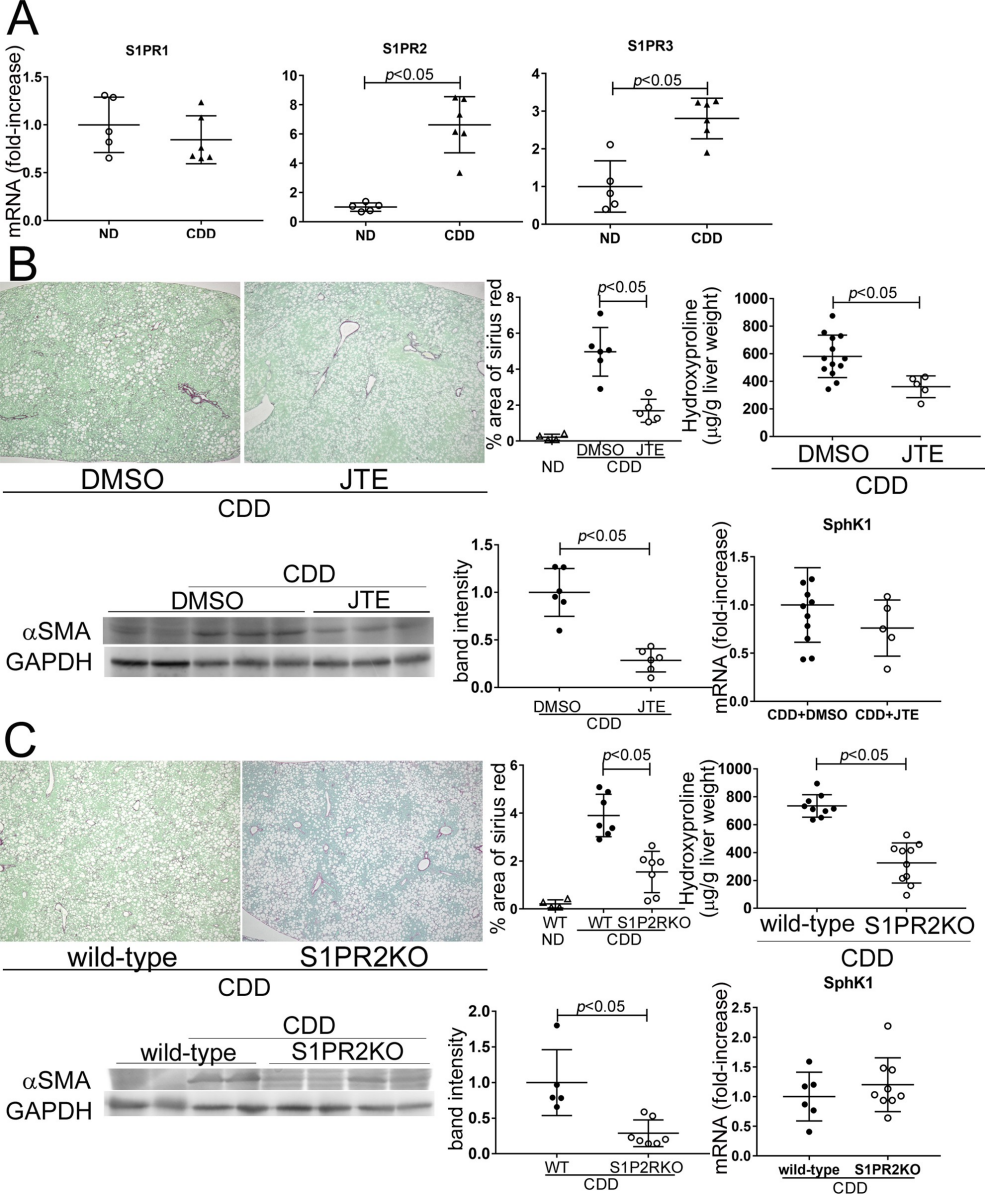
The effects of inhibition or knockout of S1PR2 on MASH. Wild-type or S1PR2KO mice were fed a normal diet or CDD for 8 weeks with or without JTE013 (JTE) treatment and were euthanized. (A) mRNA expression of S1PR1, S1PR2, and S1PR3 in the livers of CDD-fed mice was measured using **quantitative real-time RT-PCR and compared to ND-fed mice. (B, C) The effects of S1PR2 inhibition (B) or S1PR2KO (C) on liver fibrosis were determined.** Collagen deposition was assessed by Sirius Red staining (upper left panels, original magnification: 40×; graph, upper middle panel) and hydroxyproline content (upper right panels). Liver protein extracts were separated on SDS-PAGE gels, and then immunoblotting was performed with antibodies against αSMA and GAPDH (lower left panels; graph, lower middle panel). mRNA expression of SphK1 in the livers of CDD-fed mice was measured using quantitative real-time RT-PCR and compared to control mice (lower right panel). Results are representative of at least 5 independent experiments. Results are shown as the means ± SD of data collected from at least 5 independent experiments. *P < 0.05 based on a 2-tailed Student’s t-test. ND; normal diet. CDD; choline-deficient diet.

In the MASH livers of CDD-treated mice, the mRNA levels of S1PR2 and S1PR3 increased (Fig 3A). To investigate the contribution of S1P/S1PR2 to liver fibrosis, the effects of S1PR2 inhibition or knockout were examined. Mice treated with the S1PR2 inhibitor JTE013 or S1PR2KO showed decreased liver fibrosis, as demonstrated by reduced Sirius Red staining, hydroxyproline content, and αSMA expression (Fig 3B and 3C). However, the inhibition or knockout of S1PR2 did not affect lipid deposition, histological features, hepatocellular damage, or SphK1 mRNA expression although slight liver weight loss was observed in S1PR2KO mice (S3A and S3B Fig, Fig 3B and 3C). These findings suggest that S1P contributes to liver fibrosis through S1PR2 in the MASH liver and that the action of S1P on liver fibrosis occurs after the development of steatosis, hepatocellular damage, and hepatitis. Primary cultured quiescent HSCs were treated with S1P and FFA (mixture of linoleic acid and oleic acid) to examine the effects of S1P on HSCs activation. S1P treatment induced proliferation and a stellate-shaped morphology (S4A Fig), which are characteristic features of HSCs activation, whereas FFA alone did not have an effect. Similarly, S1P treatment increased αSMA expression which was further enhanced by combination therapy with FFA (S4B Fig), suggesting that S1P activates HSCs and FFA supports the effects of S1P. The induction of αSMA by S1P was inhibited by JTE013 but not by an S1PR1 inhibitor (Ex26) or S1PR3 inhibitor (CAY10444) (S4B Fig), indicating that S1P contributes to the activation of HSCs through S1PR2. The effect of S1P on macrophages was examined using peritoneal macrophages (PECs). In contrast to lipopolysaccharide (LPS) treatment, S1P did not affect morphology or TNF-α mRNA expression (S4C Fig), suggesting that S1P plays a minor role in macrophage activation.

### SphK1 expression in LSECs

SphK1 was co-localized with the LSEC marker CD31 in the MASH livers of CDD-treated mice, and treatment with TNF-α induced an elevation of SphK1 mRNA in isolated LSECs (S5A and S5B Fig). These data indicate that SphK1 is increased in LSECs in the MASH liver, suggesting that LSECs may be one of the sources of S1P. In the MASH livers of mice, the expression levels of the LSEC markers CD31, CD146, and stabilin-2 were increased compared to those in the control diet-fed mice (S5C Fig). Additionally, the mRNA expression levels of the angiogenic factors ANGP1 and ANGP2 were increased in MASH livers, whereas vascular endothelial growth factor (VEGF)A expression was comparable to that in control livers (S5D Fig). Plasma ANGP2 levels increased in MASH mice (S5D Fig). These findings suggest that angiogenesis is induced in the MASH liver and that SphK1 expression is increased in activated and capillarized LSECs, leading to S1P production.

The expression levels of CD31, CD146, and SphK1 decreased in the livers of S1PR2KO mice fed CDD (S5E Fig). Moreover, S1P treatment increased CD31 expression in primary cultured LSECs (S5F Fig). These results suggest that S1P may contribute not only to the activation of HSCs but also to the capillarization of LSECs.

### The roles of bioactive lipids other than S1P

Sphingomyelinases generates ceramides through the hydrolysis of sphingomyelin. An increase in ceramide generated by acid sphingomyelinase (ASM) in hepatocytes is involved in hepatocyte apoptosis [23]. Therefore, we examined the effects of ASM knockout on hepatocytes. Hepatocyte-specific ASMKO mice showed comparable liver fibrosis and ceramide accumulation after CDD feeding to control mice (S6A and S6B Fig), suggesting that ASM plays a minor role in ceramide elevation and MASH development. PAF and eicosanoids are generated from glycerophospholipids and function as bioactive lipids in the liver [5,6]. It has been reported that liver fibrosis induced by a highly-refined carbohydrate-containing diet is reduced in PAF receptor KO mice [24]. Lysophospholipid acyltransferase 9 [25,26] (LPLAT9, also known as lysophosphatidylcholine acyltransferase [LPCAT2]) biosynthesizes PAF from lyso-PAF [25,27]. However, LPLAT9KO mice [27] showed liver fibrosis comparable to that of wild-type mice (S6 Fig) after CDD feeding. Although PAF is altered in the CDD-induced MASH liver, it plays a minor role in MASH development.

## Discussion

In this study, we investigated the role of bioactive lipids in the progression of liver fibrosis in steatotic mice. Our data revealed that MASH livers exhibited steatosis, hepatocellular damage, angiogenesis, and fibrosis, accompanied by an increase in S1P levels in the liver. S1P stimulated the activation of HSCs and liver fibrosis via S1PR2-mediated signaling. These findings suggest novel therapeutic possibilities for inhibiting the progression of liver fibrosis in MASH.

We observed a correlation between SphK1 mRNA expression and liver fibrosis in MASH livers. In advanced fibrosis, liver steatosis was reduced (S2 Table), a condition referred to as burned-out MASH. This indicates that steatosis does not induce SphK1 induction. The inhibition or knockout of S1PR2 resulted in reduced fibrosis and angiogenesis, whereas steatosis and hepatocellular damage were comparable to those in wild-type mice after CDD feeding. The involvement of S1P is known to function in vascular development and angiogenesis [28,29]. Thus, during the transition from steatosis to hepatitis, certain factors stimulate LSECs, leading to an increase of SphK1 and S1P production. Subsequently, S1P activates both LSECs and HSCs, thereby promoting fibrosis. Among the angiogenic factors, ANGP1 and ANGP2 were upregulated in the MASH livers, whereas VEGFA expression was comparable to that in control livers. ANGP-1 contributes to vascular stability, whereas ANGP-2 stimulates remodeling and destabilization [30,31]. Treatment with a mixture of FFAs (linoleic and oleic acids) induces lipid droplets accumulation and increases triglycerides in primary cultured hepatocytes [32]. In our study, the same FFA treatment increased ANGP2 mRNA levels in primary cultured hepatocytes but not in PECs, LSECs, or HSCs (S4 Table). Moreover, ANGP1 expression increased in HSCs but not in hepatocytes after the FFA treatment, and no ANGP1 mRNA expression was detected in PECs or LSECs (S4 Table). TNF-α increased SphK1 expression in LSECs. Thus, steatosis and inflammation can trigger the initial activation and capillarization of LSECs, leading to S1P and ANGP2 production. Although S1P from activated platelets and red blood cells is a major source of plasma S1P, thrombosis was not observed in the MASH liver, indicating a minor role for S1P from activated platelets and red blood cells. In addition to the direct effects of S1P on LSECs and HSC activation, S1P also affects hepatocytes, triggering triglycerides accumulation [9], and immune cells, which may contribute to MASH development [28].

Serum and liver ceramide levels are increased in patients with MASH [33]. C16:0-ceramide is involved in hepatocyte apoptosis induced by TNF-α in mice [23] and is released into extracellular vesicles from the hepatocytes of patients with MASH [34]. Consistent with this finding, we observed an increase in ceramide levels in MASH livers. Therefore, ceramides may be involved in lipotoxicity. However, in our model, hepatocyte-specific ASMKO mice showed comparable liver fibrosis and ceramide accumulation after a CDD, suggesting that ceramides generated by neutral SM or de novo synthesis may play a role in the MASH liver. LPLAT9KO did not affect liver fibrosis after CDD feeding, suggesting that PAF plays a minor role in MASH development. LPLAT9 affects the production of eicosanoids. However, the roles of eicosanoids in the progression of MASH remain unclear. Thus, further experiments, such as using cPLA KO mice, are required.

In our MASH model, plasma FFA levels were comparable to those in control animals. However, various cells in the liver are exposed to FFAs from the gut in patient with MASLD, and the role of FFAs in MASH has been reported. For example, saturated FFAs such as PA and stearic acid directly induce cell death in cultured hepatocytes [35]. Treatment with a mixture of FFAs (linoleic and oleic acids) induced lipid deposition in primary cultured hepatocytes [32] and accelerated HSC activation by S1P. The FFA treatment alone did not result in a significant increase in collagen mRNA levels in primary cultured HSCs (1 ± 0.08 vs. 1.17 ± 0.22, p = 0.19). In human hepatic stellate cell line LX-2 cells, the addition of a mixture of oleate and palmitate acids leads to their uptake into the cells and induces the expression of pro-fibrotic genes such as αSMA, transforming growth factor-β1, and tissue inhibitor of metalloproteinase-1 [36]. Although the mechanism underlying the accelerating effect of FFAs on HSC activation remains unknown, FFAs play a role in HSCs activation. Specifically, PA (1 ± 0.48 vs. 6.48 ± 4.64, p = 0.03), but not the mixture of linoleic and oleic acids (1 ± 0.48 vs. 1.36 ± 0.84, p = 0.41), increased TNF-α mRNA expression in PECs. The FFA mixture treatment did not affect CD31 expression in LSECs (1 ± 0.46 vs. 0.92 ± 0.45, p = 0.83). In contrast, it has been reported that linoleic acid, but not oleic acid, activates endothelial cells [37]. Thus, specific FFAs may play distinct roles in various liver cells during MASH development.

In this study, the data do not provide direct evidence that S1P production in LSECs contributes to liver angiogenesis and fibrosis. Experiments using mice with conditional knockout of SphK and S1PR2 are also required to confirm the involvement of S1P from LSECs in liver fibrosis. In conclusion, our findings indicate that angiogenesis, accompanied by an increase in S1P levels, is induced in MASH livers, and that S1P promotes liver fibrosis. Therefore, targeting S1P represents a novel therapeutic strategy for treating liver fibrosis in MASH.

## Supporting information

S1 Fig CDD induced MASH in mice. Five-week-old wild-type male mice were fed a normal diet or CDD for 8 weeks and were euthanized. (A) Liver sections were stained with H-E (original magnification: 40×). (B) Body weight and liver weight were measured. (C) Hepatic lipid content was assessed by oil red O staining (left panels, original magnification: 200×) and measuring triglycerides (middle panel). Total phosphatidylcholine content in the liver was determined by LC/MS (right panel). (D) Collagen deposition was assessed by staining with Sirius Red (left panels; original magnification ×40) and by measuring the hydroxyproline content (right panel). (E) Liver protein extracts were separated on SDS-PAGE gels, and immunoblotting was performed with antibodies against αSMA and GAPDH. (F) Serum ALT levels were determined. (G) mRNA expression of TNF-α in the liver was measured using quantitative real-time RT-PCR. Results are shown as the means ± SD of data collected from at least 3 independent experiments. *P < 0.05 based on a 2-tailed Student’s t-test. ND; normal diet. CDD; choline-deficient diet.
(TIF)

S2 Fig SphK1 mRNA expression in the liver of MASLD patients. The levels of mRNA expression of SphK1 in the livers from MASLD patients was measured using quantitative real-time RT-PCR. Correlations of the SphK1 expression with the indicated values and indexes are shown. The values for Spearman’s correlation coefficient are indicated.
(TIF)

S3 Fig The effects of S1PR2 inhibitor or knockout on CDD-induced liver disease. Wild-type or S1PR2KO mice were fed a normal diet or CDD for 8 weeks with or without JTE013 (JTE) treatment and were euthanized. (A) Hepatic lipid content was assessed by oil red O staining (left panels, original magnification: 200×). Liver weight were measured (right panels). (B) Liver sections were stained with H-E (left panels, original magnification: 40×). Serum ALT levels were determined (right panels). Results are representative of at least 5 independent experiments. Results are presented as means ± SD of data collected from at least 5 independent experiments. *P < 0.05 versus control using a 2-tailed student t-test. ND; normal diet. CDD; choline-deficient diet.
(TIF)

S4 Fig S1P stimulated HSCs activation. Primary HSCs were pre-treated with or without 5 μM Ex26 (Ex), JTE013 (JTE), or CAY10444 (CAY) in serum free medium for 2 hours. Then, an FFA mixture of linoleic acid (18.8 mg/L) and oleic acid (18.8 mg/L) and/or S1P (1 μM) were added to the medium, and the cells were incubated for 4 days. (A) The morphology of the cells was observed by phase contrast microscopy (original magnification; ×200). Vitamin A autofluorescence was merged to the pictures to confirm purity of the isolated HSCs. (B) Cell protein extracts were separated on SDS-PAGE gels and immunoblotting was performed with antibodies against αSMA and GAPDH (graphs, right panels). (C) CD11b^+^ peritoneal macrophages were isolated from wild-type or the GFP mice. The cells were treated with S1P (1 μM) or LPS (50 ng/mL) for 19 hours. The morphology of the GFP^+^ cells was observed by fluorescence microscopy (left panels, original magnification; upper panels ×100 [3 different fields of view], lower panels ×400). mRNA expression of TNF-α in the wild-type cells was measured using quantitative real-time RT-PCR (right panel). Results are representative of at least 3 independent experiments. Results are presented as means ± SD of data collected from at least 3 independent experiments. *P < 0.05 versus control using a 2-tailed student t-test.
(TIF)

S5 Fig Angiogenesis was induced in the MASH liver. C57BL/6J wild-type or S1PR2KO male mice were fed a normal diet or CDD for 8 weeks and were euthanized. (A) SphK1 and CD31 were double stained with FITC-conjugated anti-SphK1 and PE-conjugated CD31 antibodies (original magnification; ×400) in the livers of the CDD-fed wild-type mice. (B) Primary cultured mouse LSECs from wild-type mice were treated with or without TNF-α (20 ng/mL) for 18 hours. mRNA expression of SphK1 in the cells was measured using quantitative real-time RT-PCR. (C) Expression of CD31, CD146, and stabilin-2 in the liver of wild-type mice was examined by immunohistochemistry with PE-conjugated anti-CD31, PE-conjugated anti-CD146, and FITC-conjugated anti-stabilin-2 antibodies. (D) mRNA expression of VEGFA, ANGP1, and ANGP2 in the livers of CDD-fed wild-type mice was measured using quantitative real-time RT-PCR and compared to ND-fed mice (left panels). Plasma ANGP2 levels were measured using ELISA (right panel). (E) CD31 or SphK1 were stained with PE-conjugated CD31 or FITC-conjugated anti-SphK1 and antibodies (original magnification; ×400). mRNA expression of CD146 and SphK1 in the livers was measured using quantitative real-time RT-PCR. (F) Primary cultured mouse LSECs from wild-type mice were treated with or without S1P (1 μM) for 18 hours. mRNA expression of CD31 in the cells was measured using quantitative real-time RT-PCR. Results are presented as means ± SD of data collected from at least 3 independent experiments. *P < 0.05 versus control using a 2-tailed student t-test.
(TIF)

S6 Fig The roles of bioactive lipids other than S1P. C57BL/6J wild-type, ASM-flox, Alb-cre/ASM flox, LPLAT9KO male mice were fed a normal diet or CDD for 8 weeks. The animals were euthanized. (A) The levels of ceramides and sphingosine were determined by LC/MS/MS. (B, C) Collagen deposition was assessed by Sirius Red staining (left panels, original magnification: 40×) and hydroxyproline content (middle panels). Results are representative of at least 4 independent experiments. Results are presented as means ± SD of data collected from at least 3 independent experiments. *P < 0.05 versus control using a 2-tailed student t-test. ND; normal diet. CDD; choline-deficient diet.
(TIF)

S1 Table Changes in the mRNA profiles of chemokines in the liver. C57BL/6J male mice were fed a normal or CDD for 8 weeks and were euthanized. Expression of the indicated mRNA variants in the liver was determined by quantitative real-time RT-PCR. Results are presented as means ± SD of data collected from at least 7 independent experiments. *P < 0.05 versus ND-treated mice using a Kruskal-Wallis-test.
(DOCX)

S2 Table The profiles of the study participants. The results are provided as means ± SDs.
(DOCX)

S3 Table Primer sequences used for quantitative real-time RT-PCR. (DOCX)

S4 Table Changes in the mRNA profiles of angiogenesis related genes in the liver cells. Primary cultured hepatocytes, peritoneal macrophages, LSECs and HSCs from C57BL/6J male mice were treated with or without fatty acids (linoleic acid (18.8 mg/L) and oleic acid (18.8 mg/L)) for 19 h. The expression of the indicated mRNA variants in the liver was determined by quantitative real-time RT-PCR. Results are presented as means ± SD of data collected from at least 3 independent experiments. *P < 0.05 versus control using a 2-tailed student t-test.
(DOCX)

S1 File Original images of Western blots in Fig 3. (TIF)

S2 File Original images of Western blots in S4 Fig. (TIF)

S3 File The ARRIVE guidlines2.0: Author checklist. (PDF)

S4 File (DOCX)

## Abbreviations

MASLD: metabolic dysfunction-associated steatotic liver dysfunction
MASH: metabolic dysfunction-accosiated steatohepatitis
CDD: choline-deficient diet
SphK: sphingosine kinase
S1P: sphingosine 1 phosphate
S1PR: S1P receptor
LSEC: liver sinusoidal endothelial cell
HSC: hepatic stellate cell
PECs: Peritoneal macrophages
FFA: free fatty acid
PAF: platelet-activating factor
PG: prostaglandin
LT: leukotriene
LPLAT9: lysophospholipid acyltransferase 9
LPCAT2: lysophosphatidylcholine acyltransferase 2
TNF-α: tumor necrosis factor-α
ANGP: angiopoietin
VEGF: vascular endothelial growth factor
HBsAg: hepatitis B surface antigen
HCVAb: hepatitis C virus antibody
AMAM2: anti-mitochondrial M2 antibody
DMSO: dimethyl sulfoxide
ALT: alanine aminotransferase
Alb-cre: albumin promoter-driven Cre recombinase
ASM: acid sphingomyelinase
LPS: lipopolysaccharide
H-E: hematoxylin and eosin
αSMA: α-smooth muscle actin
GAPDH: glyceraldehyde-3-phosphate dehydrogenase (GAPDH)
CCL: chemokine (C-C motif) ligand
CXCL: chemokine (C-X-C motif) ligand
