# Supplementary figures and images for: Sphingosine-1-phosphate promotes liver fibrosis in metabolic dysfunction-associated steatohepatitis
Source: PLoS One. 2024 May 16;19(5):e0303296. doi: 10.1371/journal.pone.0303296 (PMC11098361; doi:10.1371/journal.pone.0303296)

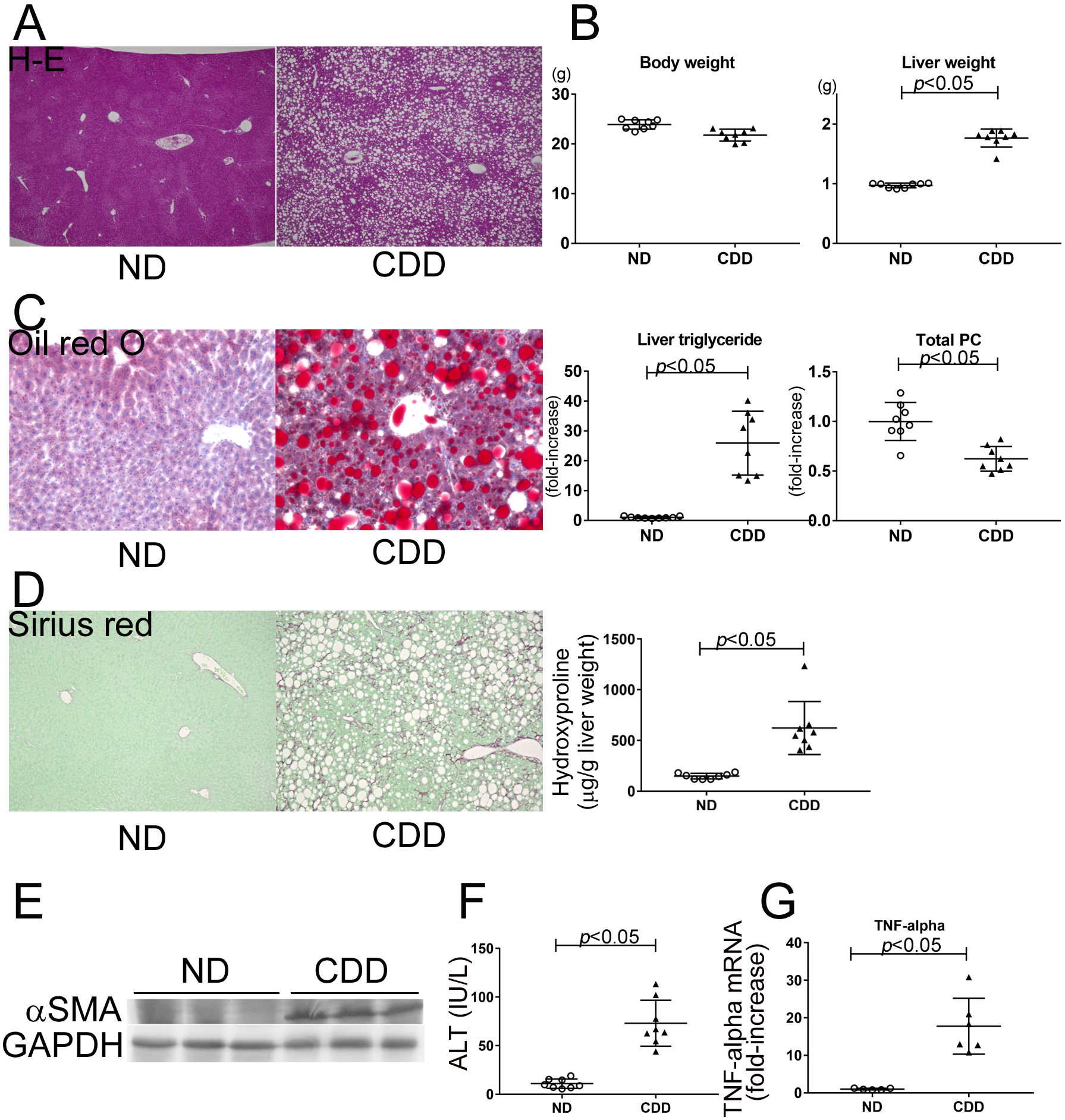

Supplement: S1 Fig — Five-week-old wild-type male mice were fed a normal diet or CDD for 8 weeks and were euthanized. (A) Liver sections were stained with H-E (original magnification: 40×). (B) Body weight and liver weight were measured. (C) Hepatic lipid content was assessed by oil red O staining (left panels, original magnification: 200×) and measuring triglycerides (middle panel). Total phosphatidylcholine content in the liver was determined by LC/MS (right panel). (D) Collagen deposition was assessed by staining with Sirius Red (left panels; original magnification ×40) and by measuring the hydroxyproline content (right panel). (E) Liver protein extracts were separated on SDS-PAGE gels, and immunoblotting was performed with antibodies against αSMA and GAPDH. (F) Serum ALT levels were determined. (G) mRNA expression of TNF-α in the liver was measured using quantitative real-time RT-PCR. Results are shown as the means ± SD of data collected from at least 3 independent experiments. *P < 0.05 based on a 2-tailed Student’s t-test. ND; normal diet. CDD; choline-deficient diet. (TIF) [file pone.0303296.s001.tif]

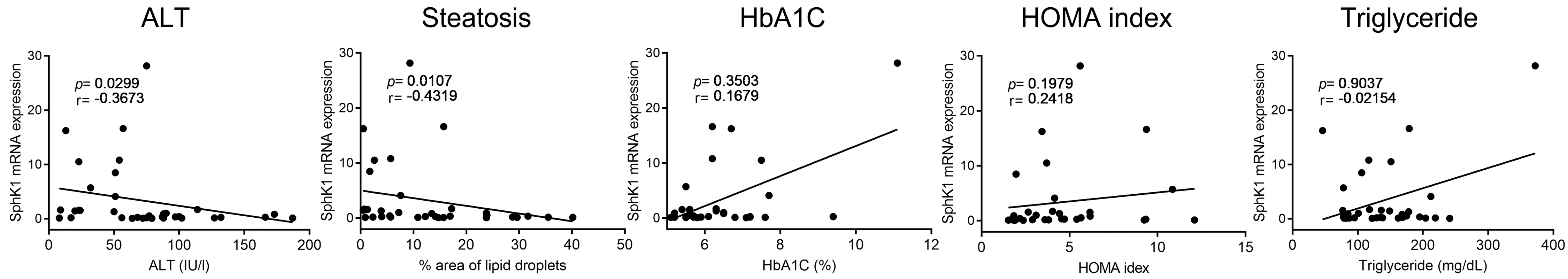

Supplement: S2 Fig — The levels of mRNA expression of SphK1 in the livers from MASLD patients was measured using quantitative real-time RT-PCR. Correlations of the SphK1 expression with the indicated values and indexes are shown. The values for Spearman’s correlation coefficient are indicated. (TIF) [file pone.0303296.s002.tif]

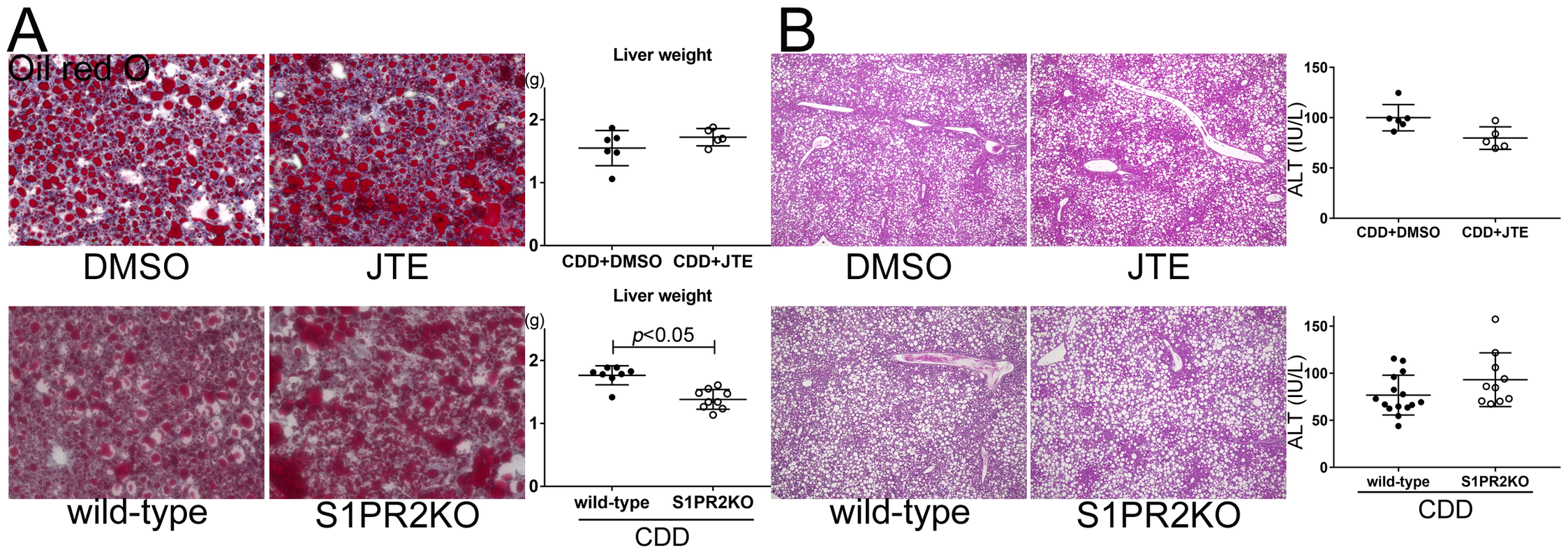

Supplement: S3 Fig — Wild-type or S1PR2KO mice were fed a normal diet or CDD for 8 weeks with or without JTE013 (JTE) treatment and were euthanized. (A) Hepatic lipid content was assessed by oil red O staining (left panels, original magnification: 200×). Liver weight were measured (right panels). (B) Liver sections were stained with H-E (left panels, original magnification: 40×). Serum ALT levels were determined (right panels). Results are representative of at least 5 independent experiments. Results are presented as means ± SD of data collected from at least 5 independent experiments. *P < 0.05 versus control using a 2-tailed student t-test. ND; normal diet. CDD; choline-deficient diet. (TIF) [file pone.0303296.s003.tif]

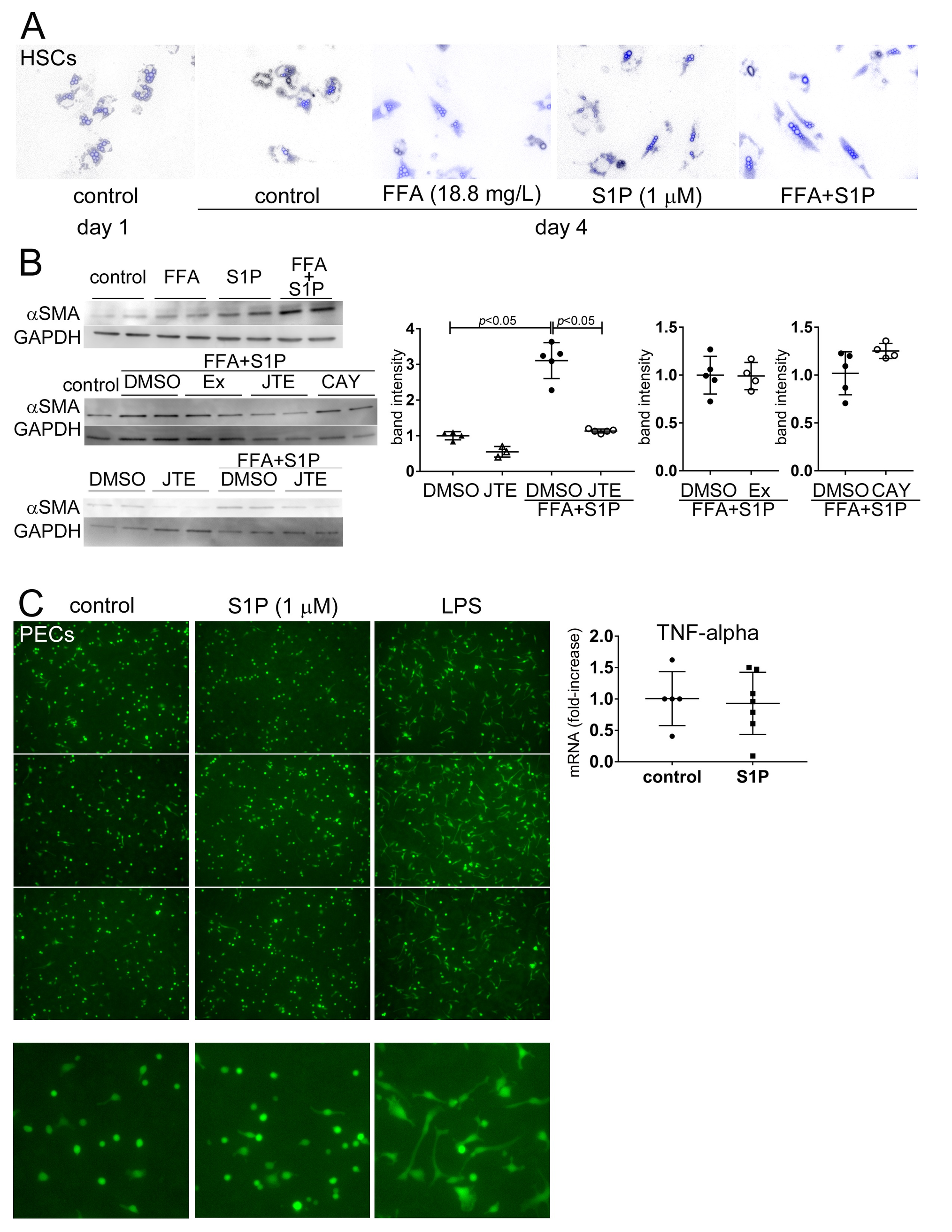

Supplement: S4 Fig — Primary HSCs were pre-treated with or without 5 μM Ex26 (Ex), JTE013 (JTE), or CAY10444 (CAY) in serum free medium for 2 hours. Then, an FFA mixture of linoleic acid (18.8 mg/L) and oleic acid (18.8 mg/L) and/or S1P (1 μM) were added to the medium, and the cells were incubated for 4 days. (A) The morphology of the cells was observed by phase contrast microscopy (original magnification; ×200). Vitamin A autofluorescence was merged to the pictures to confirm purity of the isolated HSCs. (B) Cell protein extracts were separated on SDS-PAGE gels and immunoblotting was performed with antibodies against αSMA and GAPDH (graphs, right panels). (C) CD11b+ peritoneal macrophages were isolated from wild-type or the GFP mice. The cells were treated with S1P (1 μM) or LPS (50 ng/mL) for 19 hours. The morphology of the GFP+ cells was observed by fluorescence microscopy (left panels, original magnification; upper panels ×100 [3 different fields of view], lower panels ×400). mRNA expression of TNF-α in the wild-type cells was measured using quantitative real-time RT-PCR (right panel). Results are representative of at least 3 independent experiments. Results are presented as means ± SD of data collected from at least 3 independent experiments. *P < 0.05 versus control using a 2-tailed student t-test. (TIF) [file pone.0303296.s004.tif]

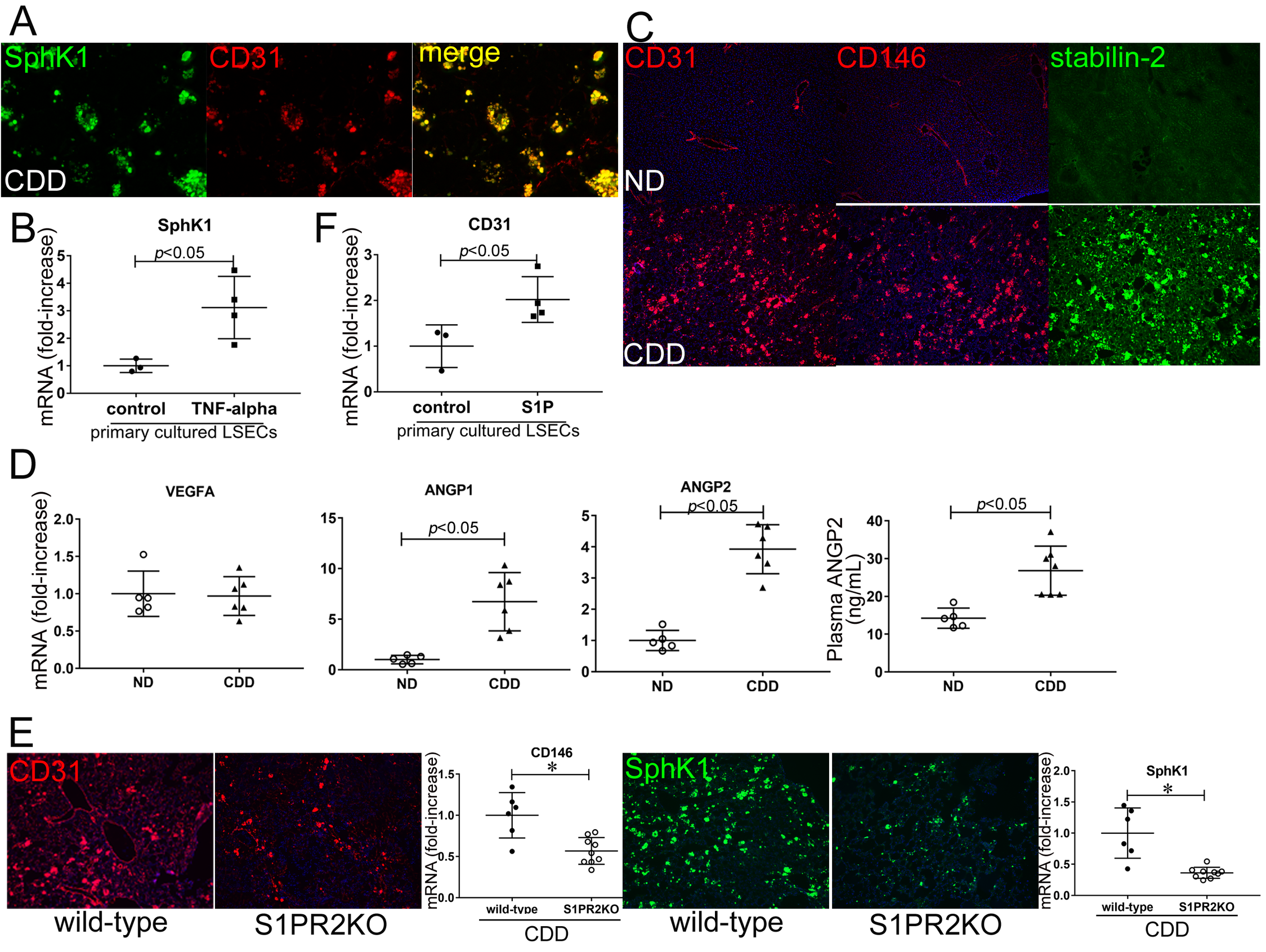

Supplement: S5 Fig — C57BL/6J wild-type or S1PR2KO male mice were fed a normal diet or CDD for 8 weeks and were euthanized. (A) SphK1 and CD31 were double stained with FITC-conjugated anti-SphK1 and PE-conjugated CD31 antibodies (original magnification; ×400) in the livers of the CDD-fed wild-type mice. (B) Primary cultured mouse LSECs from wild-type mice were treated with or without TNF-α (20 ng/mL) for 18 hours. mRNA expression of SphK1 in the cells was measured using quantitative real-time RT-PCR. (C) Expression of CD31, CD146, and stabilin-2 in the liver of wild-type mice was examined by immunohistochemistry with PE-conjugated anti-CD31, PE-conjugated anti-CD146, and FITC-conjugated anti-stabilin-2 antibodies. (D) mRNA expression of VEGFA, ANGP1, and ANGP2 in the livers of CDD-fed wild-type mice was measured using quantitative real-time RT-PCR and compared to ND-fed mice (left panels). Plasma ANGP2 levels were measured using ELISA (right panel). (E) CD31 or SphK1 were stained with PE-conjugated CD31 or FITC-conjugated anti-SphK1 and antibodies (original magnification; ×400). mRNA expression of CD146 and SphK1 in the livers was measured using quantitative real-time RT-PCR. (F) Primary cultured mouse LSECs from wild-type mice were treated with or without S1P (1 μM) for 18 hours. mRNA expression of CD31 in the cells was measured using quantitative real-time RT-PCR. Results are presented as means ± SD of data collected from at least 3 independent experiments. *P < 0.05 versus control using a 2-tailed student t-test. (TIF) [file pone.0303296.s005.tif]

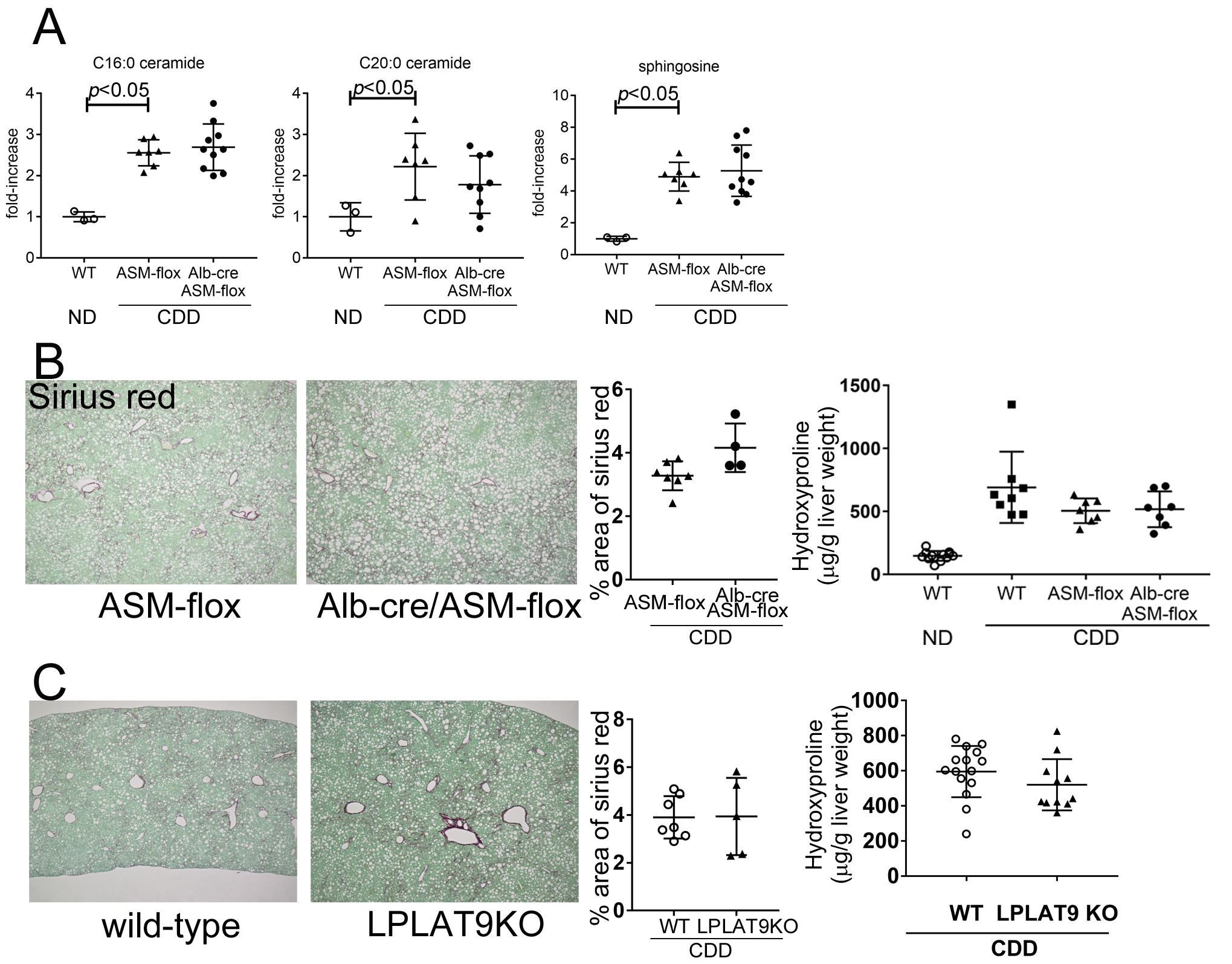

Supplement: S6 Fig — C57BL/6J wild-type, ASM-flox, Alb-cre/ASM flox, LPLAT9KO male mice were fed a normal diet or CDD for 8 weeks. The animals were euthanized. (A) The levels of ceramides and sphingosine were determined by LC/MS/MS. (B, C) Collagen deposition was assessed by Sirius Red staining (left panels, original magnification: 40×) and hydroxyproline content (middle panels). Results are representative of at least 4 independent experiments. Results are presented as means ± SD of data collected from at least 3 independent experiments. *P < 0.05 versus control using a 2-tailed student t-test. ND; normal diet. CDD; choline-deficient diet. (TIF) [file pone.0303296.s006.tif]

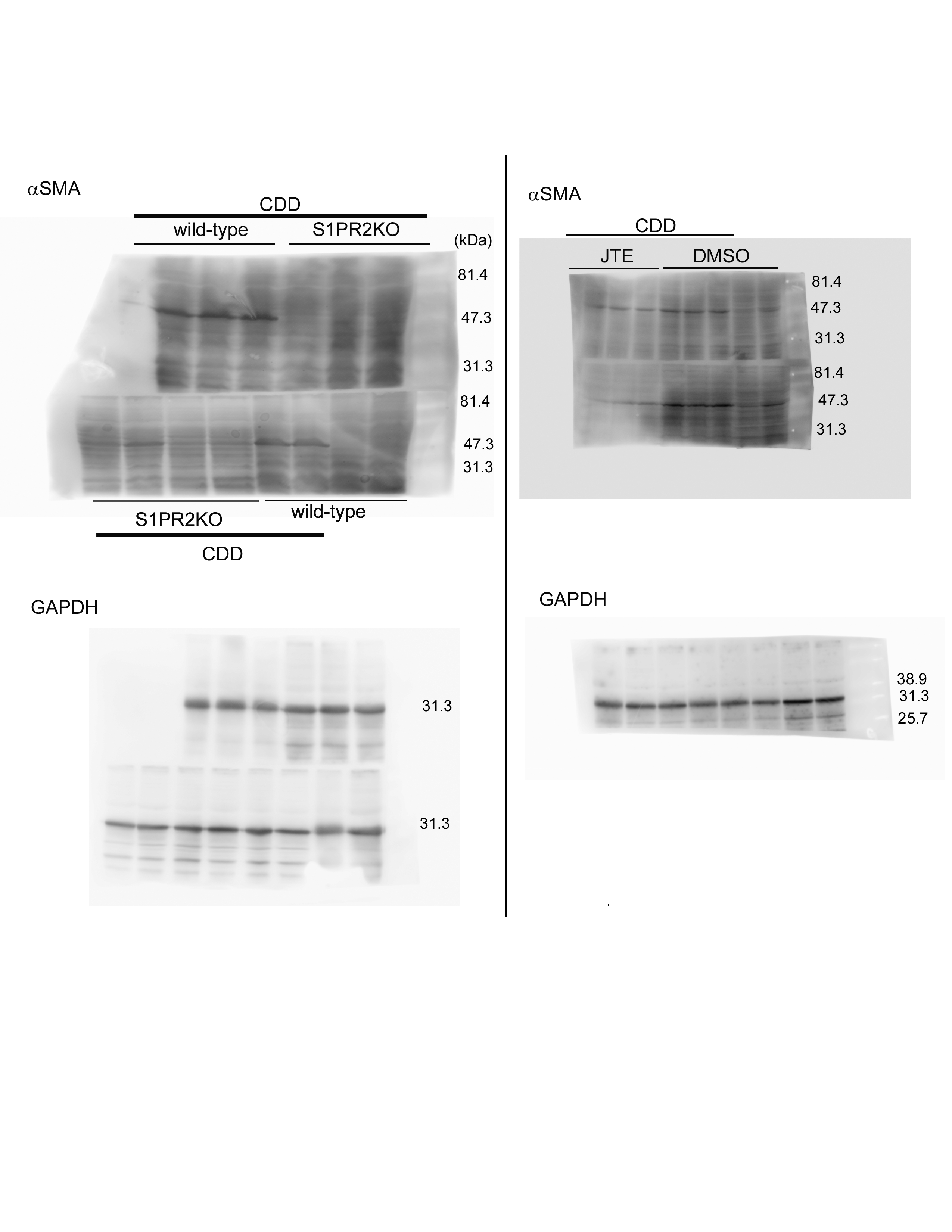

Supplement: S1 File — (TIF) [file pone.0303296.s011.tif]

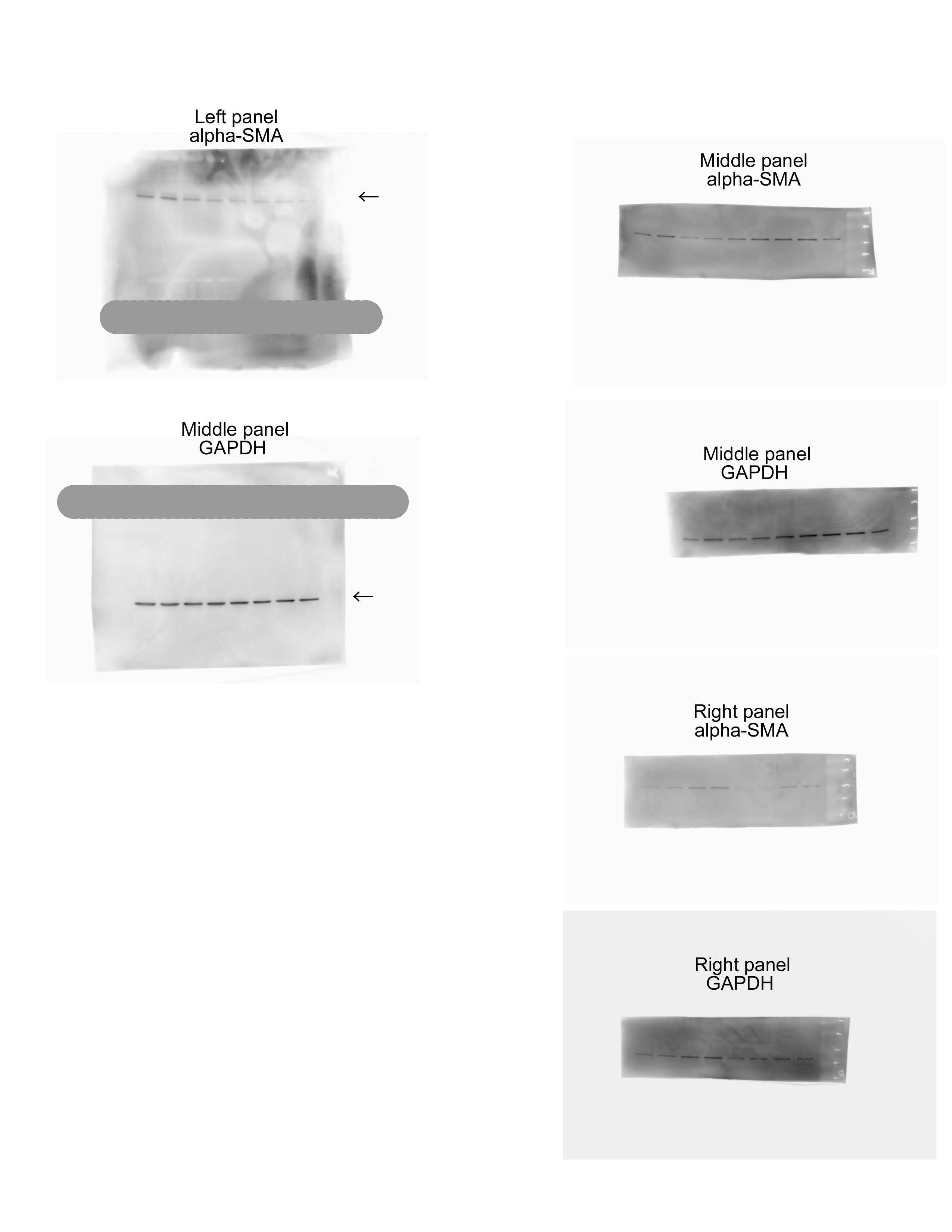

Supplement: S2 File — (TIF) [file pone.0303296.s012.tif]
