# Supplementary material for: Sphingosine-1-phosphate promotes liver fibrosis in metabolic dysfunction-associated steatohepatitis
Source: PLoS One. 2024 May 16;19(5):e0303296. doi: 10.1371/journal.pone.0303296 (PMC11098361; doi:10.1371/journal.pone.0303296)
Supplement: S1 Table — C57BL/6J male mice were fed a normal or CDD for 8 weeks and were euthanized. Expression of the indicated mRNA variants in the liver was determined by quantitative real-time RT-PCR. Results are presented as means ± SD of data collected from at least 7 independent experiments. *P < 0.05 versus ND-treated mice using a Kruskal-Wallis-test. (DOCX) [file pone.0303296.s007.docx]

| (fold-increase) | ND | CDD |
| --- | --- | --- |
| CCL2 | 1.00 ± 0.36 | 12.41± 3.04^*^ |
| CCL3 | 1.00 ± 0.47 | 20.27 ± 7.28^*^ |
| CCL4 | 1.00 ± 0.39 | 22.18 ± 6.85^*^ |
| CCL7 | 1.00 ± 0.27 | 17.78 ± 6.52^*^ |
| CCL8 | 1.00 ± 1.70 | 4.85 ± 2.66^*^ |
| CXCL1 | 1.00 ± 0.38 | 6.63 ± 2.09^*^ |
| CXCL2 | 1.00 ± 0.37 | 18.62 ± 5.93^*^ |
| CXCL3 | 1.00 ± 0.35 | 8.87 ± 2.74^*^ |
| CXCL9 | 1.00 ± 0.40 | 2.52 ± 0.92 |
| CXCL10 | 1.00 ± 0.35 | 3.41 ± 1.06^*^ |
| CXCL12 | 1.00 ± 0.17 | 1.59 ± 0.44 |
| CXCL13 | 1.00 ± 0.11 | 0.18 ± 0.11^*^ |
| CXCR4 | 1.00 ± 0.60 | 993.4 ± 840.1^*^ |
| CXCR7 | 1.00 ± 0.46 | 6.16 ± 1.76^*^ |
