# Supplementary material for: Sphingosine-1-phosphate promotes liver fibrosis in metabolic dysfunction-associated steatohepatitis
Source: PLoS One. 2024 May 16;19(5):e0303296. doi: 10.1371/journal.pone.0303296 (PMC11098361; doi:10.1371/journal.pone.0303296)
Supplement: S2 Table — The results are provided as means ± SDs. (DOCX) [file pone.0303296.s008.docx]

|  | control | MASLD | | | | |
| --- | --- | --- | --- | --- | --- | --- |
| F-stage | F0 | F0 | F1 | F2 | F3 | F4 |
| number | 5 | 3 | 13 | 6 | 3 | 5 |
| HCC / metastasis / CCC  / dysplastic nodule | 0 / 3 / 1 / 1 | 0 / 0 / 0 / 0 | 0 / 0 / 0 / 0 | 0 / 0 / 0 / 0 | 1 / 0 / 0 / 0 | 3/ 0 / 0 / 0 |
| male / female | 2 / 3 | 3 / 0 | 8 / 5 | 1 / 5 | 0 / 3 | 4 / 1 |
| age | 73.4 ± 8.8 | 52 ± 17.5 | 48.3 ± 17.3 | 46.7 ± 6.4 | 71± 12.1 | 70.4 ± 1.7 |
| NAFL / NASH |  | 1 / 2 | 1 / 12 | 0 / 6 | 0 / 3 | 0 / 5 |
| BMI | 26 ± 3.7 | 24.5 ± 5.8 | 28.8 ± 4.6 | 32.5 ± 3.9 | 27.4 ± 3.6 | 27.4 ± 3.5 |
| % area of sirius red | 1.14 ± 0.4 | 2.39 ± 2.07 | 3.26 ± 4.48 | 6.71 ± 6.32 | 4.75 ± 1.79 | 13.6 ± 3.57 |
| % area of lipid droplets | 1.11 ± 0.72 | 17.72 ± 10.55 | 18.48 ± 13.05 | 13.9 ± 7.34 | 9.57 ± 6.88 | 4.56 ± 3.83 |
| ALT (IU/l) | 15.6 ± 6.9 | 84 ± 18.3 | 100.4 ± 49.2 | 83.7 ± 29.5 | 71.7 ± 36.7 | 41.3 ± 28.5 |
| albumin (g/dl) | 3.6 ± 0.3 | 4.5 ± 0.6 | 4.3 ± 0.5 | 4.3 ± 0.5 | 4.1 ± 0.2 | 3.9 ± 0.7 |
| triglyceride (mg/dl) | 81.6 ± 53.5 | 135.3 ± 91.5 | 140.4 ± 47.7 | 139.2 ± 40.6 | 169.3 ± 47.6 | 171.5 ± 140.6 |
| HbA1C (%) | 4.9 ± 2.9 | 5.6 ± 0.3 | 6.3 ± 1.2 | 5.2 ± 2.6 | 6.8 ± 0.8 | 7.9 ± 2.2 |
| type 4 collagen 7s (ng/ml) | N. A. | 3.6 ± 0.4 | 4.6 ± 1.3 | 4.5 ± 0.8 | 6.1 ± 2.3 | 6.7 ± 1.5 |
| hyaluronan (ng/ml) | N. A. | 35.7 ± 10.7 | 65.8 ± 121.6 | 31.2 ± 16.8 | 174.7 ± 100.9 | 124.3 ± 64 |
| child-pugh classification (A / B / C) |  |  |  |  |  | 3 / 2 / 0 |
| SphK1 mRNA expression (fold-increase) | 1 ± 0.76 | 0.47 ± 0.43 | 0.42 ± 0.45 | 4.41 ± 6.82 | 2.38 ± 1.52 | 16.4 ± 8.25 |
