# Supplementary material for: Sphingosine-1-phosphate promotes liver fibrosis in metabolic dysfunction-associated steatohepatitis
Source: PLoS One. 2024 May 16;19(5):e0303296. doi: 10.1371/journal.pone.0303296 (PMC11098361; doi:10.1371/journal.pone.0303296)
Supplement: S3 Table — (DOCX) [file pone.0303296.s009.docx]

|  | Forward sequence | Reverse sequence |
| --- | --- | --- |
| Mouse | | |
| S1PR1 | CGCAGTTCTGAGAAGTCTCTGG | GGATGTCACAGGTCTTCGCCTT |
| S1PR2 | gccaacagtctccaaaacca | gagtataagccgccatggt |
| S1PR3 | TGGTGTGCGGCTGTCTAGTCAA | CACAGCAAGCAGACCTCCAGA |
| CCL2 | ATTGGGATCATCTTGCTGGT | CCTGCTGTTCACAGTTGCC |
| CCL3 | GTGGAATCTTCCGGCTGTAG | ACCATGACACTCTGCAACCA |
| CCL4 | GAAACAGCAGGAAGTGGGAG | CATGAAGCTCTGCGTGTCTG |
| CCL7 | CCTGGGAAGCTGTTATCTTCAA | TGGAGTTGGGGTTTTCATGTC |
| CCL8 | GAAGGGGGATCTTCAGCTTT | TCTTTGCCTGCTGCTCATAG |
| CXCL1 | TCTCCGTTACTTGGGGACAC | CCACACTCAAGAATGGTCGC |
| CXCL2 | TCCAGGTCAGTTAGCCTTGC | CGGTCAAAAAGTTTGCCTTG |
| CXCL3 | CAGCCACACTCCAGCCTA | CACAACAGCCCCTGTAGC |
| CXCL9 | CGGACTTCACTCCAACACAG | TAGGGTTCCTCGAACTCCAC |
| CXCL10 | TTCTGGAGGGTAACACAGTGGT | ACACCCTTTGTATCAAGTGGCA |
| CXCL12 | TGCACGGCTGAAGAACAACAACAG | TCACACCTCTCACATCTTGAGCCT |
| CXCL13 | CAGGCCACGGTATTCTGGA | CAGGGGGCGTAACTTGAATC |
| CXCR4 | GACGGACAAGTACCGGCTGC | GACAGCTTAGAGATGATGAT |
| CXCR7 | GAGGTCACTTGGTCGCTCTC | GTGTCCACCACAATGCAGTC |
| VEGFA | AAAGGCTTCAGTGTGGTCTGAGAG | GGTTGGAACCGGCATCTTTATC |
| ANGP1 | TGCAGCAACCAGCGCCGAAA | CAGGGCAGTTCCCGTCGTGT |
| ANGP2 | TCCAAGAGCTCGGTTGCTAT | AGTTGGGGAAGGTCAGTGTG |
| CD146 | AGACCGGAATTGATTGCTTG | CCCAGTACCTGAACACCTGGA |
| human | | |
| SphK1 | GGCTGCTGTCACCCATGAA | TCACTCTCTAGGTCCACATCAG |
