# Supplementary material for: Sphingosine-1-phosphate promotes liver fibrosis in metabolic dysfunction-associated steatohepatitis
Source: PLoS One. 2024 May 16;19(5):e0303296. doi: 10.1371/journal.pone.0303296 (PMC11098361; doi:10.1371/journal.pone.0303296)
Supplement: S4 Table — Primary cultured hepatocytes, peritoneal macrophages, LSECs and HSCs from C57BL/6J male mice were treated with or without fatty acids (linoleic acid (18.8 mg/L) and oleic acid (18.8 mg/L)) for 19 h. The expression of the indicated mRNA variants in the liver was determined by quantitative real-time RT-PCR. Results are presented as means ± SD of data collected from at least 3 independent experiments. *P < 0.05 versus control using a 2-tailed student t-test. (DOCX) [file pone.0303296.s010.docx]

| Primary cultured  hepatocytes | control | FFA |
| --- | --- | --- |
| VEGFA | 1.00 ± 0.32 | 0.91± 0.09 |
| ANGP1 | 1.00 ± 0.61 | 0.40 ± 0.13 |
| ANGP2 | 1.00 ± 0.30 | 1.68 ± 0.21^*^ |
| Peritoneal  macrophages | control | FFA |
| VEGFA | 1.00 ± 0.00 | 0.76 ± 0.40 |
| ANGP1 | N. D. | N. D. |
| ANGP2 | 1.00 ± 0.00 | 0.81 ± 0.97 |
| LSECs | control | FFA |
| VEGFA | N. D. | N. D. |
| ANGP1 | N. D. | N. D. |
| ANGP2 | 1.00 ± 0.30 | 0.92 ± 0.32 |
| Primary cultured  HSCs | control | FFA |
| VEGFA | 1.00 ± 0.32 | 0.46 ± 0.14^*^ |
| ANGP1 | 1.00 ± 0.37 | 1.56 ± 0.14^*^ |
| ANGP2 | 1.00 ± 0.81 | 0.57 ± 0.28 |
