# Supplementary material for: Sphingosine-1-phosphate promotes liver fibrosis in metabolic dysfunction-associated steatohepatitis
Source: PLoS One. 2024 May 16;19(5):e0303296. doi: 10.1371/journal.pone.0303296 (PMC11098361; doi:10.1371/journal.pone.0303296)
Supplement: S4 File — (DOCX) [file pone.0303296.s014.docx]

**Supplementary experimental procedures**

**Animals**

Wild-type (C57BL/6J) mice were obtained from Japan SLC (Shizuoka, Japan). The acclimatization period was 1 week. S1PR2KO mice (C57BL6 background) were obtained from Mutant mouse resource and research centers (chapel Hill, NC, USA). The transgenic mice expressing enhanced green fluorescent protein (GFP mice) were obtained from CLEA Japan, Inc. (Tokyo, Japan). The albumin promoter-driven Cre recombinase (Alb-cre) mice were purchased from Jackson Laboratory (#016832). Acid sphingomyelinase (ASM)-floxed mice were generated by UNITECH (Chiba, Japan), where a gene region containing exon 2-6 of *ASM* was flanked with loxP sites. ASM-flox mice were crossed with Alb-cre mice to delete ASM in hepatocytes. Seven ASM-flox mice and 7 Alb-cre/ASM-flox mice, all born from the same dam, were utilized. Lysophospholipid acyltransferase 9 (LPLAT9, also known as LPCAT2, Acc. NO. CDB0649K, <https://large.riken.jp/distribution/mutant-list.html>) KO mice were generated as previously reported (Ref. 25). The order of treatments and measurements was randomized, and consistent environmental conditions were maintained for animal housing and cage placement.

**Cell isolation**

Primary cultured LSEC were isolated by a nonrecirculating *in situ* collagenase perfusion of livers, cannulating through the inferior vena cava. Livers were first perfused *in situ* with a calcium-free salt solution containing 0.5 mM EGTA, followed by perfusion with a solution containing collagenase (FUJIFILM Wako Pure Chemical Corporation, Osaka, Japan). Subsequently, the liver was gently minced on a Petri dish and filtered using polyamide mesh. Hepatocytes were removed by centrifugation at 50 *g* for 1 min. The non-parenchymal cell suspension was purified by 25% and 50% Percoll (GE Healthcare, Sunnyvale, CA, USA) gradient centrifugation at 800 g for 20 min. The cells located at the 25/50% interface were collected, and LSEC were further purified using MACS cell separation columns with PE-conjugated anti-CD146 antibody and anti-PE microbeads (Miltenyi Biotec, Bergisch Gladbach, Germany). For the isolation of mouse HSCs, the liver was perfused with collagenase and pronase E (EMD Chemicals, Gibbstown, NJ, USA). Following digestion, the cell suspension was filtered through nylon mesh and purified via 8.2% Nycodenz (Axis-Shield, Oslo, Norway) gradient centrifugation. To isolate peritoneal macrophages, the mice were intraperitoneally injected with cold normal saline. Peritoneal macrophages (PECs) were purified from the lavage fluid using MACS Cell Separation Columns with CD11b microbeads (Miltenyi Biotec).

**Cell culture and treatment**

The isolated HSCs were cultured in uncoated plastic dishes with DMEM supplemented with 10% FBS and antibiotics for 12 hours. Subsequently, the cells were washed twice with PBS, and the medium was changed to serum-free RPMI 1640 containing antibiotics, with or without a mixture of linoleic acid (18.4 mg/L) and oleic acid (18.4 mg/L) (L9655, Sigma-Aldrich, St. Louis, MO, USA), and/or 1 μM S1P (human serum albumin/S1P complex, 360492P, Sigma-Aldrich), and incubated for 4 days. In some experiments, the cells were pretreated with 5 μM Ex26 (TOCRIS Bioscience, UK), JTE013, or CAY10444 (CAY10009458, CAY10444, Cayman Chemical, Ann Arbor, MI, USA) for 2 hours. The control cells were treated with bovine serum albumin. The isolated PECs were plated on uncoated dishes in RPMI-1640 with 10% FBS and antibiotics for 4 hours. After washing twice with PBS, and the medium was changed to serum free RPMI 1640 containing antibiotics, with or without 1 μM S1P or lipopolysaccharide (LPS) (50 ng/mL, L7011, Sigma-Aldrich), and incubated for 19 hours. The isolated LSEC were plated on collagen-coated dishes in RPMI-1640 containing 10% FBS supplemented with vascular endothelial growth factor (VEGF) (10 ng/mL, recombinant murine VEGF_165_, PEPROTECH, Rocky hill, NJ, USA) and antibiotics, and incubated for 12 hours. After washing with PBS twice, the cells were treated with or without mouse TNF-α (20 ng/mL, 410-MT, R&D Systems, Inc. Minneapolis, MN, USA) or 1 μM S1P for 19 hours in the same composition medium.

**Histological analysis**

The fixed tissue was sectioned into 4-μm-thick slices and stained with hematoxylin and eosin (H-E). Collagen deposits were visualized by Sirius Red staining, using a solution containing saturated picric acid, 0.1% DirectRed 80, and 0.1% FastGreen FCF. The area occupied by lipid droplets or Sirius Red positive area was quantified using ImageJ software (National Institutes of Health, Bethesda, MD, USA) and expressed as a percentage of the total section area. For immunostaining, 10-μm-thick frozen liver sections were cut using a cryostat and fixed with methanol/acetone. The sections were then stained with specific antibodies for CD31, CD146, stabilin-2 and SphK1. The antibodies used were CD31 (12-03-11-81, PE-conjugated, eBioscience), CD146 (134704, PE-conjugated, BioLegend, San Diego, CA, USA), stabilin-2 (D317-A48, Alexa Fluor 488-conjugated, MBL, Nagoya, Japan), and SphK1 (sc-365401 FITC, FITC-conjugated, Santa Cruz Biotechnology, Santa Cruz, CA, USA). Nuclei were stained with mithramycin-and 4’-6-diamindino-2-ohenylindole (DAPI). For lipid droplet staining, the frozen sections were fixed with 10% formalin and stained with Oil red O working solution (Muto Pure Chemicals, Tokyo, Japan).

**Measurement of bioactive lipids**

The levels of S1P and ceramides were determined using LC-MS/MS analysis following previously reported methods (Refs. 12, 21, 23) with slight modifications. A L-column3 C8 (3 μm, 2.0 × 100 mm, Chemicals Evaluation and Research Institute, Tokyo, Japan) was used. S1P levels in the plasma were measured using the mouse sphingosine 1 phosphate ELISA Kit (MBS094198, MyBio Source, San Diego, CA, USA). The levels of eicosanoids and PAF were determined using LC/MS analysis as previously reported (*J. Chromatogr B.* 2015; 995-996: 74-84). The levels of cholesterol ester and free cholesterol in the liver were measured using methods described in a previously report (*J Pharmacol Exp Ther.* 2015; 355: 299-307).

**ANGP2 measurement**

ANGP2 levels in the plasma were measured using mouse ANGP2 ELISA Kit (MANG20, R&D).

**Western blotting**

Protein extracts from the liver or the cells were electrophoresed and then blotted with antibodies against α-smooth muscle actin (αSMA) (ab5694, Abcam,) and glyceraldehyde-3-phosphate dehydrogenase (GAPDH) (FUJIFILM Wako Pure Chemical Corporation, Osaka, Japan). The protein bands were quantified by densitometry using Image J software. The changes were normalized based on the values of GAPDH.

**Quantitative real-time reverse transcription (qRT)-PCR**

RNA extraction from liver tissue and cultured cells, as well as reverse transcription, were performed using the RNeasy and DNase kits (Qiagen, Valencia, CA, USA) and the high-Capacity cDNA reverse transcription kit (Thermo Fisher Scientific), respectively. qRT-PCR was conducted using the SYBR Premix Ex Taq (Takara, Shiga, Japan) for mouse S1PR1, S1PR2, S1PR3, chemokine (C-C motif) ligand (CCL)2, CCL3, CCL4, CCL7, CCL8, chemokine (C-X-C motif) ligand CXCL1, CXCL2, CXCL3, CXCL9, CXCL10, CXCL12, CXCL14, CXCR4, CXCR7 VEGFA, ANGP1, ANGP2, CD146, human SphK1, (primer sequences were shown in Supplementary table 3). For mouse TNF-α, SphK1, SphK2, and 18s rRNA (mouse and human), probe and primer sets were used (Thermo Fisher Scientific, TNF-α (Mm00443258m1), SphK1 (Mm00448841g1), SphK2 (Mm00445021m1), and 18S rRNA (Hs99999901s1)). The qPCR reactions were performed using Thunderbird Probe qPCR mix (Toyobo, Tokyo, Japan) with the LightCycler 480 (Roche Applied Science) or the QuantStudio real time PCR system (Thermo Fisher Scientific). The changes were normalized based on the 18S rRNA values.

**Alanine aminotransferase (ALT) and FFA measurement**

ALT and FFA levels in the mouse plasma were measured using the transaminase CII-test Wako and NEFA C-test Wako kits, respectively (FUJIFILM Wako).

Measurement of triglycerides and phosphatidyl choline in the livers.

For triglyceride measurement, frozen liver tissues were homogenized in PBS, and methanol was added to the lysates. Lipids were extracted using the Bligh and Dyer method, and the triglyceride content was determined using the triglyceride kit L-type Wako TG M with a lipid calibrator (FUJIFILM Wako, Osaka, Japan). The levels of phosphatidylcholine in the livers were analyzed using electrospray ionization LC/MS analysis.

**Hydroxyproline measurement.**

Liver tissue was homogenized and hydrolyzed for 24 h at 110°C in 6 N HCl. The samples were oxidized with chloramine-T (Sigma-Aldrich) and incubated in Ehrlich’s perchloric acid solution. Sample absorbance was measured at 560 nm. Purified hydroxyproline (Sigma-Aldrich) was used to set a standard. Hydroxyproline content is expressed here as micrograms of hydroxyproline per gram of liver.

**Data analysis**

The principal investigators were aware of the group allocations. Those responsible for conducting the experiments, who were not principal investigators, were unaware of the group allocations. The individuals who evaluated the outcomes were unaware of the group allocations. The individuals who performed the data analysis were aware of the group allocations.
